# Impact of weight loss for depressive symptom in mid-stage patients with Parkinson’s disease: a 4-year follow-up study

**DOI:** 10.3389/fneur.2023.1306138

**Published:** 2024-01-05

**Authors:** Kanako Kurihara, Shinsuke Fujioka, Takayasu Mishima, Yoshio Tsuboi

**Affiliations:** Department of Neurology, Fukuoka University, Fukuoka, Japan

**Keywords:** Parkinson’s disease, weight loss, depression, non-motor symptoms, Zung Self-Rating Depression Scale

## Abstract

**Introduction:**

Weight loss is one of the non-motor symptoms frequently seen in patients with Parkinson’s disease (PwPD). Weight loss in PwPD is known to be negatively associated with motor and other non-motor symptoms and has been shown to influence the prognosis of PD. In this study, we followed weight change over a 4-year period in PwPD at a single institution and investigated the relationship between weight change and patients’ motor and non-motor symptoms.

**Methods:**

PwPD who visited our hospital from January 2018 to December 2022 were enrolled. Body weights were measured at two points in 2018 (at the start of observation, ‘baseline’) and 2022 (at the end of observation, ‘end date’). In addition, motor symptoms, disease severity, cognitive function, and psychiatric symptoms were evaluated during the same period, and the relationship with weight loss was examined.

**Results:**

Data of 96 PwPD were available for a 4-year follow-up. At baseline, the mean age was 65.7 ± 10.0 years, the mean disease duration was 6.8 ± 4.0 years, and the mean Hoehn and Yahr stage was 2.4 ± 0.7. Among them, 48 patients (50.0%) had a weight loss of ≥5% from baseline (weight loss group; mean loss was 6.6 ± 2.9 kg). The weight loss group was older (*p* = 0.031), had a lower Mini-Mental State Examination (MMSE) at baseline (*p* = 0.019), a significantly lower body mass index (*p* < 0.001), and a higher Zung Self-Rating Depression Scale (SDS) (*p* = 0.017) at the end date. There was a negative correlation (γ = −0.349, *p* < 0.001) between weight change and age, a positive correlation (γ = 0.308, *p* = 0.002) between weight change and MMSE at baseline, and a negative correlation (γ = −0.353, *p* < 0.001) between weight change and SDS at the end date. Age-adjusted correlations showed a final negative correlation (γ = −0.331, *p* = 0.001) between weight change and SDS. MMSE and age-adjusted correlations showed a low negative correlation (γ = −0.333, *p* = 0.001) between weight change and SDS at the end date.

**Conclusion:**

Weight loss in PwPD in mid-stage was more likely with increasing age, and ≥ 5% weight loss was associated with worsening depression. Further research is needed regarding the significance of weight loss in PwPD.

## 1 Introduction

Parkinson’s disease (PD) is a chronic progressive neurodegenerative disease characterized by motor symptoms including bradykinesia, resting tremor, rigidity, and postural instability; however, it has been shown that non-motor symptoms precede motor symptoms and significantly affect patients’ quality of life over time (1). Weight loss is one of the most frequent non-motor symptoms, occurring in 48.6–55.6% of patients with Parkinson’s disease (PwPD) (2, 3), and is a symptom that can be present even before the onset of motor symptoms; it can occur at any stage of the disease from early to advanced (4, 5). Such weight loss deserves more attention because it is independently associated with decreased quality of life (6) and mortality (7). Weight loss has also been reported to affect cognitive decline (8), orthostatic hypotension (9), and the appearance of dyskinesia (10). Determinants of weight loss in PwPD are multifactorial; known risk factors include age, duration of disease, total Movement Disorder Society Unified Parkinson’s Disease Rating Scale (MDS-UPDRS), and Hoehn & Yahr (HY) stage (2, 11). To date, few studies have longitudinally followed up on weight loss and clinical symptoms over a long period of time at the same institute. In this study, we investigated the association between weight loss and other factors in PwPD attending our hospital over a 4-year period.

## 2 Materials and methods

### 2.1 Protocol approval

All patients were > 20 years old. This study was performed in accordance with the Declaration of Helsinki and approved by the Fukuoka University Medical Ethics Review Board (U20-04-001).

### 2.2 Patients and study design

This study was conducted as a single-center, longitudinal study in Japan. PwPD received treatment at the Department of Neurology, Fukuoka University Hospital, from January 2018 to December 2022. All patients were examined by a movement disorder specialist and were diagnosed with clinically established PD or probable PD according to the International Parkinson and Movement Disorder Society diagnostic criteria (12).

Demographic and background information such as body weight, body mass index (BMI), age, sex, age at disease onset, duration of disease, dyskinesia, olfactory decline, and hallucinations were extracted from each patient’s medical records. Levodopa-equivalent daily dose (LEDD) was calculated from their medications according to standard assessments (13). Motor symptoms were evaluated by a movement disorder specialist using the HY stage (14) and the MDS-UPDRS part III (15). Cognitive function was assessed with the Japanese version of the Montreal Cognitive Assessment (MoCA) (16, 17) and the Mini-Mental State Examination (MMSE). Depression and apathy were assessed using the Zung Self-Rating Depression Scale (SDS) (18) and the Apathy Scale (19). Patients’ quality of life was assessed using the Parkinson’s Disease Questionnaire-8 (PDQ-8) (15), and summary indexes (PDQ-8 SI) were calculated (20). The 9-symptom Wearing-OFF Questionnaire (WOQ-9) (21) was used to evaluate the phenomenon of “wearing off.” In this study, patients were considered to have worn off if they had two or more positive symptoms on the WOQ-9 and if they improved with dopaminergic therapy. The presence of constipation was diagnosed using the Rome III criteria (22). We performed these evaluations during the patients’ ON state. Data collected in 2018 (baseline) were compared to data collected in 2022 (end date). All participants were measured by their body weight (in kilograms) at baseline and the end date. We weighed all of our patients using the same scale as TANITA Co. (DC-320) at the outpatient clinic in the morning. The shoes, jackets, and heavy clothing were removed, and pockets were emptied at the time of weighing. According to the difference resulting between the baseline and the end-date body weight, subjects were divided into a weight loss group and a weight stable/gain group. There is no uniform definition of significant weight loss. Some previous studies (2, 11, 23) adopted a weight loss of ≥5% as the definition of weight loss for study periods ranging from 6 months to 10 years. Similar to these studies, patients with a weight loss of ≥5% were defined as having significant weight loss in this study.

### 2.3 Statistics

BMI, age, age at onset, disease duration, LEDD, HY stage, UPDRS part III, MMSE, MoCA, SDS, Apathy Scale, and PDQ-8-SI were analyzed by the Mann–Whitney U-test between the two groups of PwPD with and without ≥5% weight loss. Changes in HY stage, UPDRS part III, MMSE, MoCA, and SDS over 4 years were analyzed by the Mann–Whitney U-test. Sex, wearing off, dyskinesia, hallucinations, olfactory decline, and constipation between the two groups were analyzed by the chi-square test. Correlation coefficients between weight change and age, MMSE, MoCA, and SDS in 2022 and 2018 were analyzed using Pearson’s correlation coefficient. The correlations were interpreted as follows: 0.9–1 indicated a very high correlation; 0.7–0.9 indicated a high correlation; 0.5–0.7 indicated a moderate correlation; 0.3–0.5 indicated a low correlation; and 0–0.3 indicated little to no correlation (24). The correlations between weight change and MMSE, MoCA, and SDS at baseline and end date were analyzed using partial correlation coefficients after controlling for age. All *p*-values <0.05 were considered statistically significant. Data were analyzed by SPSS v.26 (SPSS Inc., Chicago, IL, United States).

## 3 Results

There were 222 PwPD for whom data were collected in 2018. Of these, 96 patients were still attending in 2022, for whom data could be collected. Table 1 shows the clinical characteristics of the patients and comparisons between the two groups: (i) weight loss of ≥5% and (ii) weight stable/gain. There were 37 men and 59 women, with mean age 65.7 ± 10.0 years, mean disease duration 6.8 ± 4.0 years, mean HY stage 2.4 ± 0.7, and mean UPDRS Part III 27.3 ± 12.2. Forty-eight patients (50.0%) had a weight loss of ≥5% (mean 6.6 ± 2.9 kg) (Table 1).

**Table 1:** Comparison between ≥5% body weight loss and weight stable/gain groups at baseline and end date.

	2018 (baseline)			2022 (end-date)		
	Weight loss (*n* = 48)	Weight stable or gain (*n* = 48)	*p*-value	Weight loss (*n* = 48)	Weight stable or gain (*n* = 48)	*p*-value
Sex, male	19 (36.6)	18 (37.5)	0.834	–	–	–
Age, years	67.9 ± 8.5	63.4 ± 11.2	0.031	71.9 ± 8.5	67.4 ± 11.2	0.031
Age at onset, years	60.8 ± 9.4	56.8 ± 11.6	0.092	–	–	–
Duration, years	7.1 ± 4.0	6.5 ± 4.0	0.503	11.1 ± 4.0	10.5 ± 4.0	0.503
Body weight, kg	56.9 ± 12.9	57.1 ± 11.8	0.660	50.3 ± 11.7	58.6 ± 13.0	0.001
BMI, kg/m^2^	23.3 ± 3.9	22.8 ± 3.9	0.435	20.5 ± 3.5	23.3 ± 4.1	<0.001
LEDD, mg	476.9 ± 309.4	548.0 ± 393.6	0.521	747.1 ± 379.0	805.9 ± 395.6	0.446
HY stage	2.5 ± 0.6	2.5 ± 0.8	0.968	2.8 ± 0.7	2.8 ± 0.8	0.932
UPDRS III	25.9 ± 9.8	28.8 ± 14.5	0.409	30.6 ± 11.5	30.2 ± 14.2	0.704
MMSE	27.9 ± 1.9	28.4 ± 1.9	0.019	27.7 ± 3.1	28.0 ± 2.7	0.440
MoCA	24.4 ± 3.3	25.2 ± 3.3	0.084	23.6 ± 4.5	24.5 ± 4.6	0.174
SDS	40.7 ± 10.2	41.3 ± 9.1	0.803	47.0 ± 9.1	42.7 ± 9.1	0.017
Apathy Scale	NA	NA	NA	13.6 ± 6.8	13.2 ± 7.4	0.698
PDQ-8 SI	15.5 ± 15.8	15.5 ± 16.5	0.845	26.2 ± 20.2	23.5 ± 16.0	0.678
Wearing OFF	19^*1^ (41.3)	20^*2^ (42.6)	0.903	20 (41.7)	29 (60.4)	0.101
Dyskinesia	12 (2.1)	9 (18.8)	0.459	20 (41.7)	19 (39.6)	0.835
RBD	24 (50.0)	16 (33.3)	0.098	19 (39.6)	24 (50.0)	0.305
Hallucination	9 (18.8)	6 (12.5)	0.399	14 (29.2)	9 (18.8)	0.232
Olfactory decline	27 (56.3)	18 (37.5)	0.066	23 (47.9)	15 (31.3)	0.095
Constipation	27 (56.3)	21^*3^ (47.7)	0.414	29 (60.4)	26 (54.2)	0.536

BMI, Body mass index; LEDD, Levodopa equivalent daily dose; HY, Hoehn & Yahr; UPDRS, Unified Parkinson’s Disease Rating Scale; MMSE, Mini-Mental State Examination; MoCA, The Montreal Cognitive Assessment; SDS, Self-Rating Depression Scale; PDQ-8 SI, The Parkinson’s Disease Questionnaire-8 Summary Index; RBD, REM sleep behavior disorder; All data are presented as mean ± standard deviation. NA: not available. All data are presented as mean ± standard deviation or n (%). ^*^1: *n* = 46, ^*^2: *n* = 47, ^*^3: *n* = 44.

The weight loss group was significantly older (*p* = 0.031) with a lower MMSE (*p* = 0.019) in 2018 and had a significantly lower BMI (*p* < 0.001) and higher SDS (*p* = 0.017) at the end date (Table 1).

**Figure 1:**
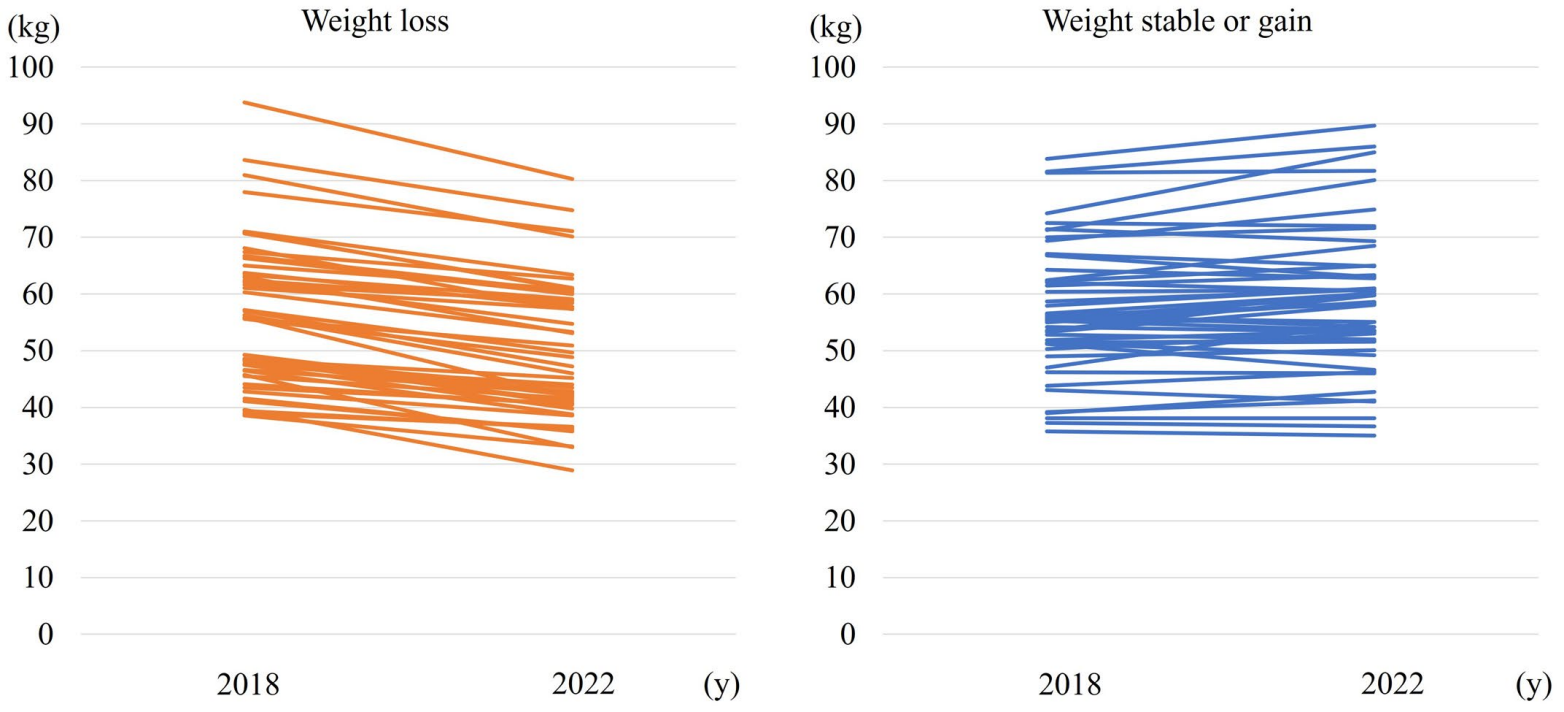
Weight change over 4 years in the 5% body weight loss and weight stable/gain groups.

Figure 1 shows weight change over 4 years in the weight loss and weight stable/gain groups.

Table 2 shows a comparison of changes in the HY stage, UPDRS Part III, MMSE, MoCA, and SDS between the weight loss and weight stable/gain groups. The weight loss group showed a significantly greater change in SDS (*p* = 0.018).

**Table 2:** Mean change between ≥5% body weight loss and weight stable/gain groups.

	Weight loss (*n* = 49)	Weight stable or gain (*n* = 47)	*p*-value
Change in body weight, kg	−6.6 ± 2.9	1.4 ± 3.3	<0.001
Change in HY stage	0.3 ± 0.6	0.3 ± 0.7	0.997
Change in UPDRS Part III	4.9 ± 10.7	1.2 ± 12.0	0.050
Change in MMSE	0 ± 2.2	−0.5 ± 2.3	0.188
Change in MoCA	−0.8 ± 3.1	−0.7 ± 4.2	0.482
Change in SDS	6.6 ± 10.0	1.5 ± 8.3	0.018

UPDRS, Unified Parkinson’s Disease Rating Scale; MMSE, Mini-Mental State Examination; MoCA, The Montreal Cognitive Assessment; SDS, Self-Rating Depression Scale; All data are presented as mean (standard deviation) or n (%).

**Figure 2:**
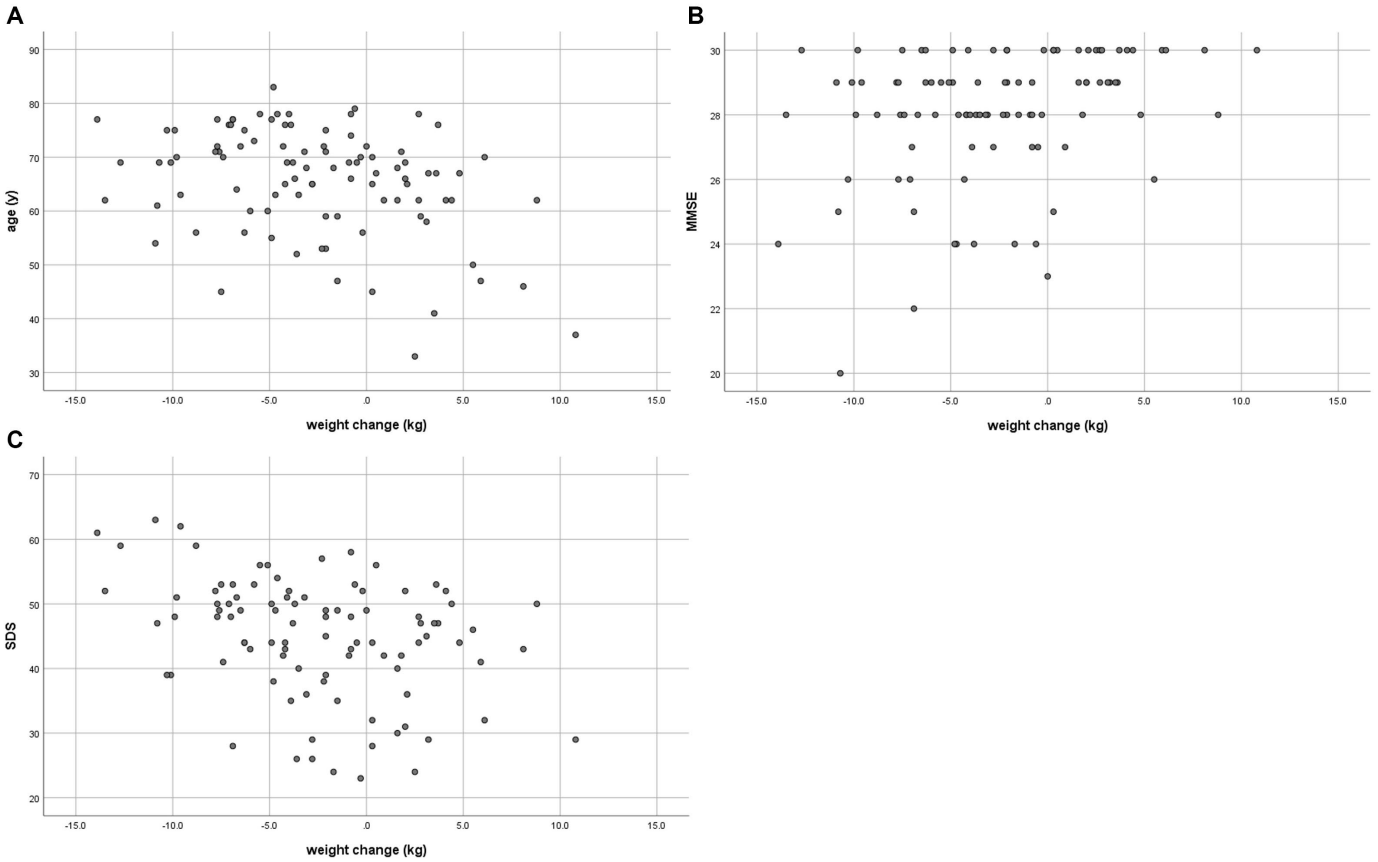
Correlation between weight change and **(A)** age in 2018, **(B)** MMSE in 2018, and **(C)** SDS in 2023.

The correlations between weight change and age, MMSE, MoCA, and SDS between baseline and end date are shown in Table 3 and Figure 2.

**Table 3:** Pearson product–moment correlation coefficient between weight change and other variables.

Year	2018 (baseline)				2022 (end date)		
Weight change vs.	Age	MMSE	MoCA	SDS	MMSE	MoCA	SDS
γ	−0.349	0.308	0.274	−0.123	0.213	0.238	−0.353
P-value	<0.001	0.002	0.007	0.231	0.037	0.020	<0.001

MMSE, Mini-Mental State Examination; MoCA, The Montreal Cognitive Assessment; SDS, Self-Rating Depression Scale.

A low negative correlation (γ = −0.349, *p* < 0.001) was found between weight change and age. There was a low positive correlation (γ = 0.308, *p* = 0.002) with MMSE at baseline and a low negative correlation (γ = −0.353, *p* < 0.001) between weight change and SDS at the end date. Age-adjusted correlations showed a low negative correlation (γ = −0.331, *p* = 0.001) between weight change and SDS at the end date (Table 4). MMSE and age-adjusted correlations showed a low negative correlation (γ = −0.333, *p* = 0.001) between weight change and SDS at the end date (Supplementary Table S1).

**Table 4:** Partial correlation coefficient between weight change and other variables after controlling for age.

Year	2018 (baseline)			2022 (end-date)		
Weight change vs.	MMSE	MoCA	SDS	MMSE	MoCA	SDS
γ	0.211	0.152	−0.171	0.135	0.102	−0.331
*p*-value	0.040	0.141	0.097	0.193	0.327	0.001

MMSE, Mini-Mental State Examination; MoCA, The Montreal Cognitive Assessment; SDS, Self-Rating Depression Scale.

## 4 Discussion

Similar to the present study, when using criteria for significant weight loss of ≥5% in PwPD, previous reports have shown that weight loss is associated with age, disease duration, total UPDRS, and HY stage (2, 11, 23). In our study, the weight loss group was significantly older, but there was no significant difference in disease duration or severity of the disease.

Of particular note in this study is the following: the weight loss group had a significantly higher SDS at the end date. Weight change was also negatively correlated with the SDS at the end date, and there were similar results for partial correlation coefficients after controlling for age or MMSE (Table 4, Supplementary Table S1). There was no difference in SDS between the two groups at baseline, suggesting weight loss in PD causes depression, although depressive symptoms and weight loss may interact with each other. Few reports have examined depression and weight loss in PD; one study reported that PwPD with a 3% weight loss showed a significant association with depression (11) and that comorbid depression affected those with a lower BMI in male PwPD (25). An association between depression and malnutrition was reported in PwPD (26). With regard to depression and weight changes, although the patient background is different from this study, it was reported that 40% of depressed patients gained weight and 30% lost weight (27), and that there were differences in activity in several brain regions in response to food stimuli between groups with increased and decreased appetite (28). The present study suggested that weight loss can cause subsequent depression; however, the mechanism by which weight loss causes depression in PD is multifactorial and cannot be determined. We need to be mindful of weight loss as it may cause complications in PwPD and depression (29).

In this study, MMSE was lower in the weight loss group at baseline, and weight change and MMSE were positively correlated. However, partial correlation coefficients after controlling for age showed no correlation. Several reports show that cognitive decline is a factor affecting weight loss in PD (3, 30), and weight changes in early PD increase cognitive decline (8). However, another study did not observe this correlation (31). Although age was a major factor in the current study, further investigation is needed to determine whether cognitive impairment is a risk for weight loss. In this study, there were no significant correlations between weight loss and quality of life or dyskinesia, which has been shown in previous studies (6, 7, 32). Our study and previous studies have shown that weight loss in PwPD could affect the worsening of motor and non-motor symptoms and, in part, quality of life and mortality (6, 7, 33, 34).

The primary limitation of this study was that it involved a single center and was small-scale. Second, it did not take into account each patient’s physical activity or nutritional status. It is reported that PwPD with weight loss increased their energy intake but decreased their activity (30); therefore, further study including data on dietary intake and physical activity is needed. Third, the differentiation of the weight gain group was not considered. In fact, there is evidence that patients with weight gain may follow different trajectories (35). Future studies should consider the fact that the number of participants in our weight gain group was not large: only 17 patients. Hence, it may be worthwhile to conduct more comprehensive investigations into this particular subgroup in future research.

To date, most weight loss studies in PwPD have focused on the early stages of the illness (35, 36), and little is known about the weight loss in the mid-stage that contributes to the alternation of the motor and non-motor symptoms. Our study suggested that in mid-stage PD, a significant weight loss of ≥5% influences psychiatric symptoms, particularly depression. We believe that regular assessments of body weight and nutritional status and intervention if needed should receive more attention in PD management. Previous studies have shown that many PwPD are at risk of malnutrition (37); therefore, they may need to be better assessed for weight changes and nutritional deficiencies. Intervention may be necessary for weight loss in early or mid-stage PwPD, but the establishment of evidence for its efficacy needs to be awaited in future. Dietitians should also be considered members of multidisciplinary PD teams in collaborative PD assessment and nutritional interventions. However, the clinical relevance of underweight and malnutrition in PD needs further investigation. In addition, at present, there is no dietary intervention specific to PwPD, and it is necessary to investigate what kind of diet influences disease progression and prognosis.

## Data availability statement

The original contributions presented in the study are included in the article/Supplementary material, further inquiries can be directed to the corresponding author.

## Ethics statement

The studies involving humans were approved by Fukuoka University-Medical Ethics Review Board. The studies were conducted in accordance with the local legislation and institutional requirements. Written informed consent for participation was not required from the participants or the participants’ legal guardians/next of kin because this research is a retrospective study using existing medical information, the research protocol has been made public, and research subjects have been assured of refusal.

## Author contributions

KK: Conceptualization, Data curation, Formal analysis, Methodology, Writing – original draft. SF: Data curation, Writing – review & editing. TM: Data curation, Writing – review & editing. YT: Conceptualization, Data curation, Supervision, Writing – review & editing.
