# Supplementary material for: Impact of weight loss for depressive symptom in mid-stage patients with Parkinson’s disease: a 4-year follow-up study
Source: Front Neurol. 2024 Jan 5;14:1306138. doi: 10.3389/fneur.2023.1306138 (PMC10796778; doi:10.3389/fneur.2023.1306138)
Supplement: Supplementary file 1 [file Table_1.docx]

Supplemental Table 1. Partial correlation coefficient between weight change and Self-rating Depression Scale after controlling for Mini-Mental State Examination and age.

| Year | 2018 (baseline) | 2022 (end date) |
| --- | --- | --- |
| Weight change vs. | SDS | SDS |
| γ | -0.184 | -0.333 |
| P-value | 0.075 | 0.001 |

SDS: Self-rating Depression Scale
